# Veil-Based Collector Device Enabling Self-Collected Cervicovaginal Sampling for Site-of-Care Primary HPV-Based Cervical Cancer and Sexually Transmitted Infections Screening: A Pilot Feasibility Study in Romania

**DOI:** 10.3390/diagnostics16172765

**Published:** 2026-08-28

**Authors:** Madalina Ciuhodaru, Alina-Mihaela Calin, Bogdan-Florentin Nițu, Claudia Simona Cambrea, Ioana Denisa Popa, Cristian Bucsineanu, Ralph-Sydney Mboumba Bouassa, Juval Avala Ntsigouaye, Vincent Vernet, David Sebaoun, Franck Chaubron, Laurent Bélec

**Affiliations:** 1 Faculty of Medicine, Grigore T. Popa University of Medicine and Pharmacy, 700115 Iasi, Romaniabucsineanu.cristian21@gmail.com (C.B.); 2 Faculty of Medicine and Pharmacy, Dunarea de Jos University, 800017 Galati, Romania; 3 Department of Dermatology, Faculty of General Medicine, Ovidius University of Constanta, 900527 Constanta, Romania; 4 Department of Infectious Diseases, Faculty of General Medicine, Ovidius University of Constanta, 900527 Constanta, Romania; 5 Happyfamilygyn SRL, Street Jupiter, No. 10, 900698 Constanta, Romania; 6 Institut du Savoir Montfort, Montfort Hospital, Ottawa, ON K1K 0T2, Canada; 7 Department of Family Medicine, Faculty of Medicine, University of Ottawa, Ottawa, ON K1H 8M5, Canada; 8 Ecole Doctorale Régionale d’Afrique Centrale en Infectiologie Tropicale, Franceville 876, Gabon; juvalavala@gmail.com; 9 Laboratoire Labio, 13090 Aix-en-Provence, France; vvernet@labio.fr (V.V.);; 10 SF Biotech SAS, 13090 Aix-en-Provence, France; 11 Laboratory of Virology, Hôpital Européen Georges Pompidou, Assistance Publique-Hôpitaux de Paris (AP-HP), 75015 Paris, France; 12 Faculté de Médecine, Université Paris Cité, 75006 Paris, France

**Keywords:** veil, female genital secretions, self-collection, multiplex PCR, sexually transmitted infections, high-risk human papillomavirus, Romania

## Abstract

**Background/Objectives**: High-risk human papillomavirus (HR-HPV) causes cervical cancer, but other sexually transmitted infections (STIs) act as possible cofactors. We herein evaluated the feasibility, logistics, and epidemiology of a community-based screening program in Romania using an innovative veil-based self-sampling device and a digital platform. **Methods**: Adult women self-collected genital secretions using the Vaginal Veil Collector V-Veil UP2™ device (V-Veil-Up Production SRL, Pitesti, Romania). A digital platform managed registration and results. Dry impregnated veils were transported at ambient temperature via standard courier to an accredited French laboratory for molecular testing using in parallel the Allplex™ HPV HR Detection assay (Seegene, Seoul, Republic of Korea), detecting 14 HR-HPVs, and the Allplex™ STI Essential Assay (Seegene), detecting 7 major pathogens causing STIs [*Chlamydia trachomatis*, *Neisseria gonorrhoeae*, *Mycoplasma genitalium*, *Trichomonas vaginalis*, *Mycoplasma hominis*, *Ureaplasma urealyticum*, *Ureaplasma parvum*]. **Results**: Among 960 included women (mean age 41.3 years), technical success was high: 97.7% of samples yielded valid results for HR-HPV and 97.4% for STIs. Sample stability at ambient temperature eliminated cold-chain requirements. Overall, 17.2% of women were positive for HR-HPV and 26.7% for an STI. The most frequent genotypes were HPV-68 (3.0%), HPV-16 (2.9%), and HPV-31 (2.3%); *Ureaplasma parvum* (24.0%) was the most prevalent bacterial pathogen. The 18–29 age group exhibited the highest risk for HR-HPV (22.2%) and STIs (36.8%). Multivariate analysis revealed strong biological associations: *Chlamydia trachomatis* was independently associated with overall HR-HPV (aOR: 4.52; 95% CI: 1.26–16.11) and multiple HR-HPV infections (aOR: 13.96; 95% CI: 3.70–52.64). *Mycoplasma hominis* and *Ureaplasma* species were also independently associated with HR-HPV (aOR: 1.83 and 1.67, respectively) or nonvaccine types (aOR: 2.75 and 5.03, respectively). **Conclusions**: Veil-based self-sampling combined with digital logistics is highly feasible, scalable, and overcomes geographical barriers. The marked association between *Chlamydia trachomatis*, *Mycoplasma hominis*, *Ureaplasma* species, and HR-HPV strongly advocates for integrated molecular co-testing models in public health screening programs.

## 1. Introduction

In Eastern Europe, including Romania, the burden of cervical cancer remains significantly high, primarily driven by persistent infection with high-risk human papillomavirus (HR-HPV) [1,2,3,4,5,6]. This region generally exhibits some of the highest incidence and mortality rates for cervical cancer across Europe, which is often attributed to suboptimal organized screening programs, low participation rates among eligible women, and limited HPV vaccination coverage [1]. For example, Romania specifically has been reported to have among the worst cervical cancer indicators in the European Union, often cited as triple the European average than in other European countries [1,7,8]. A review of European cervical cancer screening policies explicitly notes that while many European countries have made progress, Romania continues to record the highest incidence and mortality rates in the entire region. Indeed, while Romania’s incidence rate is up to 2.5 times higher than the European Union average, the mortality rate can be even higher, up to 4 times [9]. This crisis is driven by persistent systemic barriers, low public awareness, fragmented policies, and suboptimal screening participation below 20% [9,10]. Organized screening programs suffer from underfunding, poor infrastructure, and a lack of functional public health tracking or registries [9]. Both routine Pap smear participation and HPV vaccination rates remain critically low among eligible populations [9,11]. According to GLOBOCAN 2024 estimates, every year in Romania, 3308 women receive a diagnosis of cervical cancer and 1743 die from the disease [12]. In 2012, the Romanian government established a national cervical cancer screening program, providing free HPV testing and cervical precancerous treatment for all women 25–64 years old referred by a program-registered general practitioner [13]. However, the continuum of care, including retesting and follow-up for women with HR-HPV positivity or detected precancerous lesions, remains restricted to those with health insurance coverage [13]. Despite the initiation of the national cervical cancer screening program, participation remains low, especially among women from the minority Roma and other ethnic groups [13,14,15]. Consequently, the high prevalence of HR-HPV infection and cervical disease burden underscores the urgent need for innovative and more effective screening strategies to reach women outside conventional clinic-based programs [13]. The introduction of self-sampling methods, as explored in this study, is a critical step toward overcoming these barriers and improving early detection and prevention efforts in this vulnerable population.

Self-collection of vaginal samples, based on non-invasive and easy-to-perform procedures, for the detection of HPV DNA constitutes an interesting alternative to increase the participation of hard-to-reach women in screening programs [16,17,18,19,20,21,22]. Veil-based self-collection of female genital secretions for the detection of HR-HPV and pathogens causing sexually transmitted infections (STIs) constitutes an attractive non-invasive and easy-to-perform method to increase the participation of women in cervical cancer and STI screening programs [23,24,25,26].

The aim of this study was to evaluate under real-life conditions the feasibility, logistics, and epidemiology of a community-based screening program for HPV and STIs in Romania among adult women using an innovative veil-based self-sampling device and a digital platform. This study also aimed to assess the molecular epidemiology of circulating HPV and STI pathogens, as well as the interactions between HR-HPV and STI pathogens as potential cofactors.

## 2. Materials and Methods

### 2.1. Study Population

Adult (≥18 years) women from public or private gynecologic centers throughout Romania were prospectively invited to participate in 2025 to the pilot study.

Inclusion criteria were: (i) female participants aged 18 to 65 years who were sexually active; (ii) individuals providing voluntary written informed consent to participate and willingness to perform self-sampling; (iii) capability to access and navigate the digital self-testing platform for account registration and receipt of laboratory results; and (iv) ability to understand and comply with study protocols and follow-up recommendations. Exclusion criteria were: (i) current pregnancy or post-partum period (<8 weeks) at the time of sampling; (ii) active menstruation on the day of sample collection; (iii) history of total hysterectomy (removal of the cervix) or invasive cervical cancer treatment; (iv) use of topical vaginal medications, douches, or spermicides within 48–72 h prior to self-collection to prevent specimen dilution or PCR inhibition; and (v) cognitive, severe psychiatric, or medical conditions that prevent informed consent or self-sampling execution.

If a participant was actively menstruating, sampling was postponed until at least 2–3 days post-menstruation. Participants were advised to refrain from sexual intercourse for 48 h prior to sampling to ensure optimal yield of host cervical–vaginal cells. No special genital washing or disinfection was required immediately before insertion, as standard baseline vaginal flora and cervical–vaginal secretions were required for accurate diagnostic testing.

### 2.2. Genital Sample Collection and Processing

Genital samples were obtained using a veil-based self-sampling kit (Vaginal Veil Collector V-Veil UP2™ device, V-Veil-Up Production SRL, Pitesti, Romania; https://hpv-veil.com/) along with the instructions for use (IFU), as described previously [23,25].

The Vaginal Veil Collector V-Veil UP2™ is a patented, class I, CE-marked medical device, specifically designed for self-collection, which contains a non-woven hydrophilic polyethylene material—the veil—and an applicator that helps insert it into the vaginal cavity [21]. The device is listed by UNITAID as a self-sampling device [26]. The vaginal veil safely and gently catches and retains the female genital secretions, thus harvesting cells, proteins, and nucleic acids (DNA/RNA). Through its composition, the veil device does not absorb the liquids, but rather adsorbs the genital’s biochemical components, thereby allowing for their full desorption after adequate elution of the collected biological material. Along with its special biomaterial composition, what makes the veil original is its large external surface [23], allowing it to easily adsorb the maximum amount of proteins and nucleic acids and further release them efficiently during the elution phase according to the established concentration gradient [27].

The veil is particularly well accepted by women across various cultural settings—such as Central Africa, the Sahel region, and Europe [23,25]. Self-collection using a non-invasive, soft-mesh veil device significantly reduces the physical discomfort, fear, and modesty concerns frequently associated with invasive speculum-based clinical examinations or rigid swabs. This approach provides a discreet, private sampling option that respects individual modesty and cultural sensitivities surrounding pelvic examinations.

Two kit formats were used for self-collection at home or in-clinic: the V-Veil UP2™ KIT 1.3, containing one individually wrapped Vaginal Veil Collector V-Veil UP2™, one wrapped UP2 Retrofitter™ Tube, and one IFU, all packed in eco-friendly packaging; and the V-Veil UP2™ KIT 4.3, containing one individually wrapped Vaginal Veil Collector V-Veil UP2™, one wrapped UP2 Retrofitter™ Tube, one safety bag complying with the rules for the delivery of biological substances category B (UN3373) and one IFU, all packed in eco-friendly packaging. The Vaginal Veil Collector V-Veil UP2™ impregnated for genital collection is placed in the UP2 Retrofitter™ tube, as the primary tube. The self-collection procedure packages V-Veil UP2™ KIT 1.3 and V-Veil UP2™ KIT 4.3 contain IFU in the Romanian language, with exemplifying images for each step to follow, including digital elements such as a QR code that leads to a video that exemplifies the steps to follow in images, with text and sound, also targeting people with hearing or vision impairments.

To respect participant autonomy, sample collection was offered through a flexible dual-track distribution model rather than a mandatory centralized pathway. For distribution, participants could receive the self-sampling package via a web portal or directly from a healthcare provider. Afterwards, collected samples could then either be sent personally by courier to the laboratory using a prepaid air waybill (AWB) or handed over at a local collection point. Local medical collection points maintained their standard operational procedures without protocol interference—some actively followed up with patients for sample collection, whereas others simply provided the kits.

At health centers or upon request, healthcare professionals could provide instructions or assist with self-sampling, using an instruction leaflet translated into Romanian. Finally, all participants performed the self-sampling at home or in a private place at health centers without external assistance, by referring only to the graphic guidance.

All impregnated dry vaginal veils were delivered by UPS in a safety bag at ambient temperature through regular logistics to the reference lab (Laboratoire Labio, Aix-en-Provence, France). Samples were kept stored at +4 °C until analysis.

This study utilized the secured Gyntest.bio digital platform for self-testing (https://labio.gyntest.com/en/ accessed on 9 July 2026) to streamline patient registration, where essential demographic data were recorded, and to enable direct email reporting of lab results to participants. Because the Gyntest.bio platform and its collaborating partner, Laboratoire Labio, are hosted and operate out of Aix-en-Provence, France, diagnostic data and relevant workflow processing were routed through France. Data transfer and handling were strictly restricted to necessary processing activities directly related to participant registration and diagnostic reporting. All operations complied with applicable General Data Protection Regulation (GDPR) guidelines for cross-border health data processing, ensuring full data encryption, confidentiality, and restricted access limited solely to authorized project personnel and the clinical laboratory.

### 2.3. Extraction and Molecular Testing

Samples were eluted in 5 mL of Tris-EDTA (TE) buffer (1X), pH 8.0, for molecular biology (AppliChem GmbH, Otto Weg, Darmstadt, Germany), and vortexed. Molecular testing of HR-HPV and STIs was carried out after automatic nucleic acid extraction of eluted samples (300 µL) with the STARMag 96 × 4 Universal Cartridge Kit (Seegene, Seoul, Republic of Korea), using the Seegene STARlet extractor (Seegene). HR-HPV detection was realized on 100 µL of extracted DNA using the CE IVD-marked multiplex one-step real-time PCR Allplex™ HPV HR Detection assay (Seegene), detecting 14 HR-HPVs (HPV-16,-18, -31, -33, -35, -39, -45, -51, -52, -56, -58, -59, -66 and -68), according to the manufacturer’s instructions. Seven major pathogens causing STIs, including 3 bacteria [*Chlamydia trachomatis* (CT), *Neisseria gonorrhoeae* (NG), *Mycoplasma genitalium* (MG)], 1 protozoan parasite [*Trichomonas vaginalis* (TV)] and commensal mycoplasma [*Mycoplasma hominis* (MH), *Ureaplasma urealyticum* (UU), *Ureaplasma parvum* (UP)], were detected on 100 µL of extracted DNA by the multiplex real-time PCR CE IVD-marked Allplex™ STI Essential Assay (Seegene), according to the manufacturer’s instructions. The DNA amplification and the genotyping process were carried out on the CFX96™ real-time PCR instrument (Bio-Rad, Marnes-la-Coquette, France). Samples, extraction, and PCR reagent setups were controlled with Seegene Launcher IVD (Seegene), and final PCR results were automatically analyzed using Seegene Viewer IVD software version 3.33 (Seegene). The Laboratoire Labio was accredited by the Comité Français d’Accréditation (COFRAC) according to the ISO 15189:2022 norm for molecular biology [28].

### 2.4. Post-Analytical Advice

The results were accessible on the secured website through participants’ personal accounts. Participants who tested positive for HR-HPV or STIs were scheduled for appropriate gynecological care. Thus, diagnostic reports clearly outlined necessary follow-up steps, providing specific clinical recommendations based on detected pathogens. Individuals testing positive for HR-HPV were advised to schedule a cervical cytology or colposcopy examination in accordance with national screening guidelines, while those with identified STIs were provided targeted antibiotic or antiviral therapy prescriptions alongside partner notification guidance. Referrals to collaborating gynecological clinics were made available through the platform to ensure prompt medical intervention, minimize loss to follow-up, and answer patient queries regarding their diagnosis.

### 2.5. Statistical Analysis

Statistical analysis was performed using GraphPad Prism version 8.4.2 (GraphPad Software, Inc., San Diego, CA, USA). The basic demographic background data (age) as well as the study location of eligible participants were analyzed to evaluate the feasibility and applicability of the online screening approach. Means and standard deviations (SDs) were calculated for quantitative variables and proportions for categorical variables. Pearson’s χ^2^ or Fisher’s exact tests were used for categorical variables and the non-parametric Mann–Whitney U-test or Kruskal–Wallis’ rank sum test for quantitative variables. Logistic regression models using univariate and multivariate analyses were performed to determine the association of independent variables with HR-HPV type-specific infections (i.e., genital infection with HR-HPV, vaccine HR-HPV, nonvaccine HR-HPV), age, inclusion site, and STIs. All statistically significant variables (*p* < 0.05) in the univariate analysis were included in a multivariate logistic regression analysis. The crude Odds ratio (cOR) and adjusted Odds ratio (aOR) were calculated, where appropriate, along with their 95% CI. Finally, a risk factor was defined as an independent variable yielding in the univariate analysis a cOR strictly greater than “1” with a *p*-value less than 0.05. An aOR strictly greater than “1” with a *p*-value less than 0.05 defined a risk factor in multivariate analysis. For categorical variables included in the logistic regression analyses, one category was selected as the reference (REF) group; cORs and aORs with their 95% CIs were estimated for the other categories relative to the reference category.

### 2.6. Ethical Statement

This study was conducted in accordance with the Ethical Guidelines of the World Medical Association Declaration of Helsinki and approved by the Institutional Review Boards and Ethics Committees of the Elena Doamna Gynecology Clinic, Iasi, Romania, of the Grigore T. Popa University of Medicine and Pharmacy, Iasi, Romania, and of the Ovidius University of Constanta, Constanta, Romania. This study is considered research with a direct individual benefit, since virological diagnosis of HR-HPV and the essential STIs was given to the participants with adapted care. Informed written consent was obtained from all participants, including consent to publish this study.

## 3. Results

### 3.1. Study Specimens

**Figure 1:**
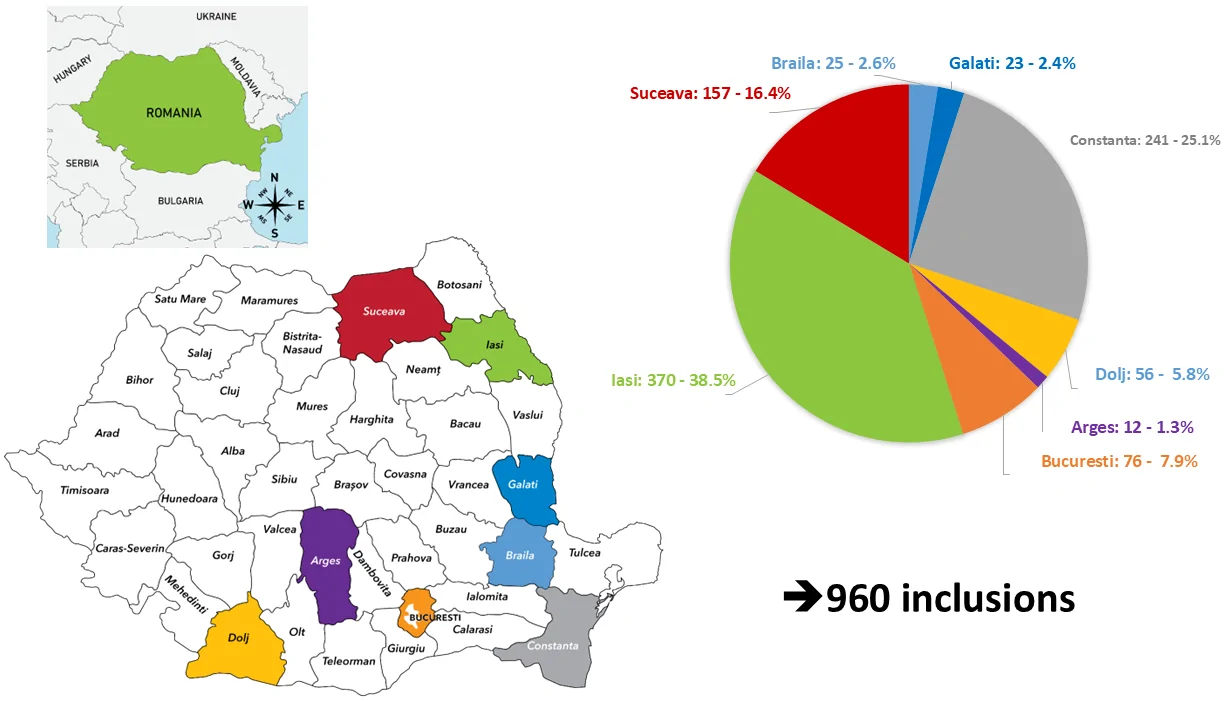
Geographic distribution of the 960 study participants recruited across Romanian counties.

The total number of adult women participating in the veil-based self-sampling protocol across all study locations in Romania was 960 (Figure 1). The highest number of participants came from the county of Iași (colored green), accounting for 370 inclusions, which represents approximately 38.5% of the total. The second-highest inclusion site was Constanța (colored gray), with 241 inclusions or about 25.1% of the total. Suceava (colored red) was the third-largest contributor, providing 157 inclusions, making up approximately 16.4% of the total. The county of București (colored light orange) contributed 76 inclusions, which is about 7.9% of the total. Dolj (Craiova, colored orange) reported 56 inclusions, equivalent to about 5.8% of the total. The remaining counties included Brăila (colored light blue) with 25 inclusions (about 2.6%), Galați (colored blue) with 23 inclusions (about 2.4%), and Argeș (Pitești, colored purple) with 12 inclusions (about 1.3%).

**Figure 2:**
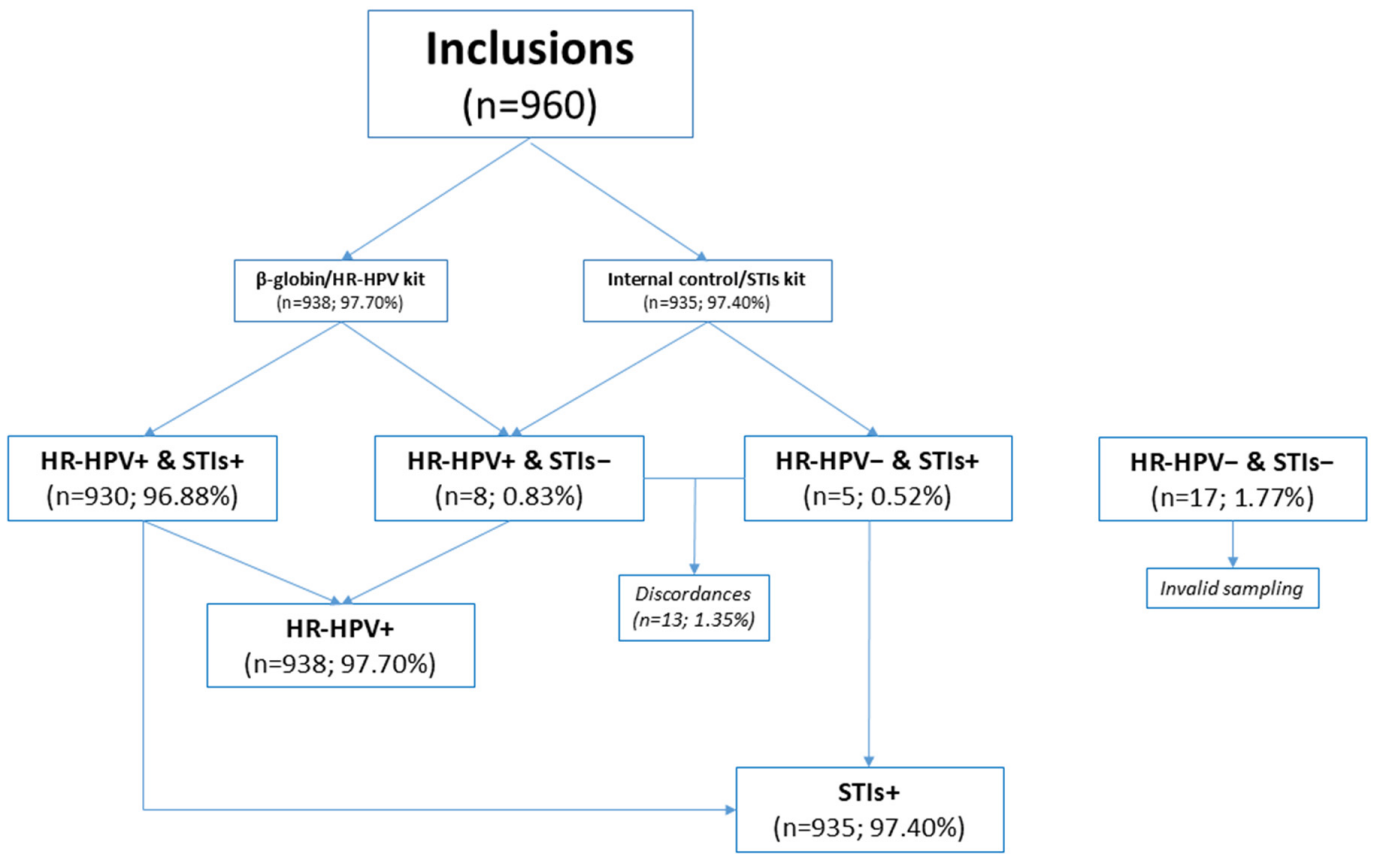
Flow chart of self-sampling inclusions and results for the β-globin housekeeping gene serving as an internal control for the Allplex™ HPV HR Detection kit (Seegene, Seoul, Republic of Korea) and for the Allplex™ STI Essential kit (Seegene).

The participant inclusion flow, sample validity, and molecular testing outcomes for HR-HPV and STI are summarized in Figure 2.

From the 960 specimens collected at inclusion, 943 (98.2%) showed internal control positivity either for the HR-HPV kit (*n* = 938; 97.7%) or the STI kit (*n* = 935; 97.4%). A total of 930 specimens (96.8%; 96.9%) showed internal control positivity for both kits. In total, 938 women showed valid β-globin internal control for HR-HPV molecular detection, equating to 97.70% of the total inclusions, and 935 women showed positive internal control for STI detection, which is 97.39% of the total. The genital sample specimens can be classified as follows: (i) Valid samples (*n* = 943; 98.23%) showing valid β-globin internal control results when tested for HR-HPV and/or valid internal control when tested for STIs, yielding three main categories: HR-HPV+ and STIs+, including 930 women who tested positive for both HR-HPV and STIs (96.88% of the total inclusions); HR-HPV+ and STIs-, including 8 women who tested positive for HR-HPV but negative for STIs (0.83% of the total); and HR-HPV- and STIs+, including 5 women who tested negative for HR-HPV but positive for STIs (0.52% of the total). (ii) Invalid samples (*n* = 17; 1.77%) showing invalid internal control results for both HR-HPV and STI molecular detection.

Table 1 depicts age and distribution of HPV types and essential STIs in the study participants according to their inclusion sites in Romania.

### 3.2. HPV Detection and Genotypes

**Figure 3:**
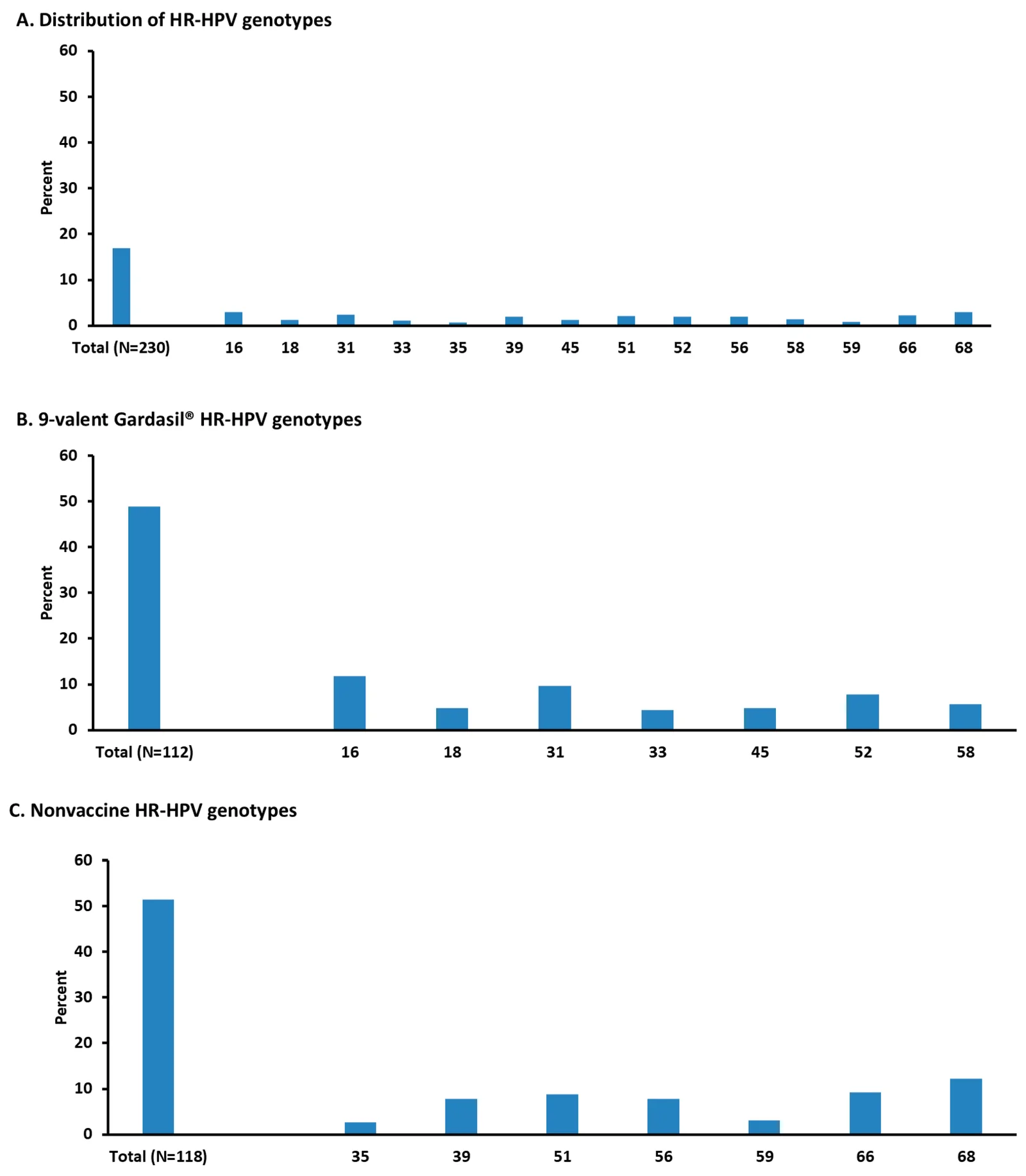
HR-HPV genotype distribution according to vaccine and nonvaccine genotype categories (**A**). The overall HR-HPV genotype distribution (**B**). Genotypes included in the 9-valent Gardasil^®^ vaccine, and (**C**) nonvaccine HR-HPV genotypes.

The distribution of HR-HPV genotypes is presented in Figure 3. Overall, 230 positive HR-HPV DNA cases were observed in 17.2% (161/938) of participants (mean age: 41.0 ± 11.6 years). The most frequent genotypes were HPV-68 (3.0%; *n* = 28), HPV-16 (2.9%; *n* = 27), HPV-31 (2.3%; *n* = 22), HPV-66 (2.2%; *n* = 21), and HPV-51 (2.1%; *n* = 20), followed by HPV-39 (1.9%; *n* = 18), HPV-52 (1.9%; *n* = 18), HPV-56 (1.9%; *n* = 18), HPV-58 (1.4%; *n* = 13), HPV-18 (1.2%; *n* = 11), HPV-45 (1.2%; *n* = 11), HPV-33 (1.0%; *n* = 10), HPV-59 (0.7%; *n* = 7), and HPV-35 (0.6%; *n* = 6). Single HR-HPV infection was present in 12.4% (116/938) of participants, whereas 4.8% (45/938) showed multiple HR-HPV genotypes (mean: 2.5; range 2 to 5).

A total of 938 samples were positive for the β-globin internal control, of which 230 HR-HPV cases were detected. Among them, 16.5% (38/230) of HR-HPV genotypes were targeted by Gardasil-4^®^ (Merck & Co. Inc., NJ, USA) in 24 (2.5%) participants, whereas 48.7% (112/230) of HR-HPV genotypes were targeted by Gardasil-9^®^ in 90 (9.6%) women. Nonvaccine genotypes represented 51.3% (118/230) of all HR-HPV detected.

### 3.3. STI Detection and Distribution

**Figure 4:**
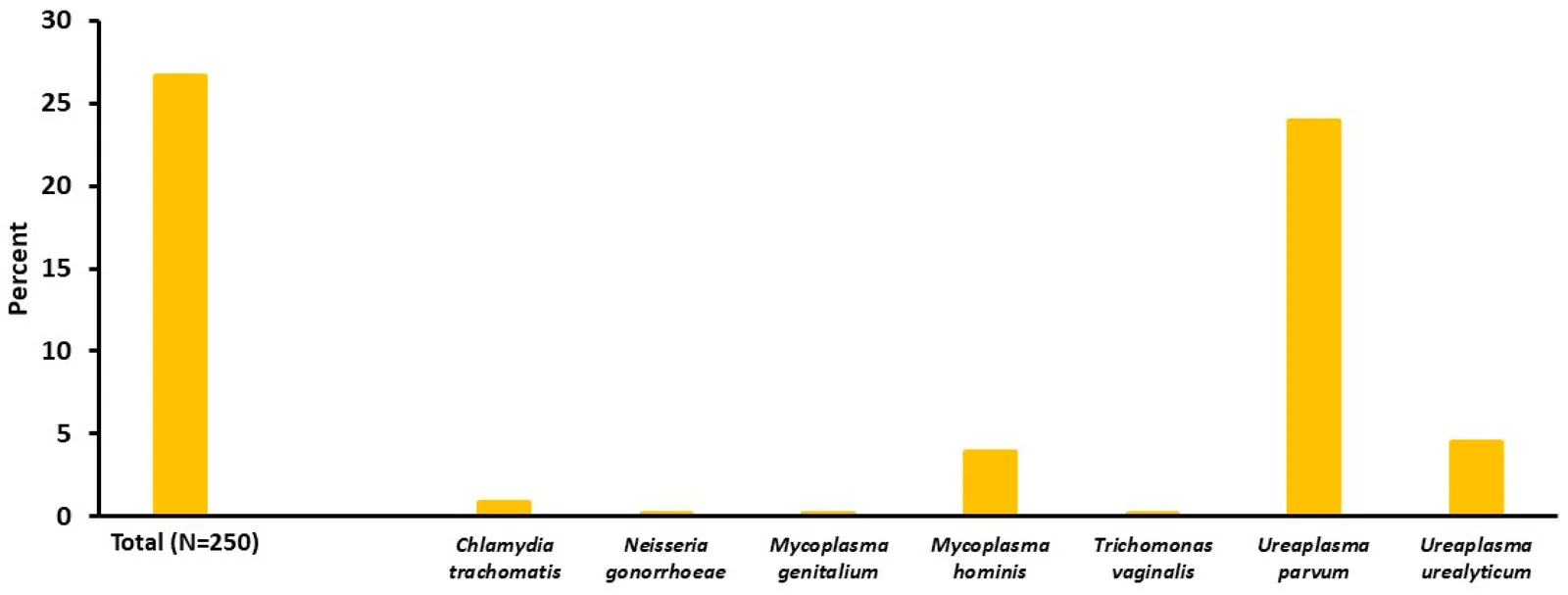
Distribution of STIs detected in genital samples from 960 volunteer adult participants recruited throughout Romania, using the multiplex one-step real-time PCR assay Allplex™ STI Essential Assay (Seegene, Seoul, Republic of Korea). A total of 935 samples were positive for the internal control, of which 250 STIs were detected.

At least one sexually transmitted pathogen was detected in 26.7% (250/935) of participants (mean age: 38.6 ± 11.5 years), as shown in Figure 4. STIs included UP (24.0%, *n* = 232), MH (3.9%, *n* = 38), UU (4.5%, *n* = 43), CT (0.9%, *n* = 9), TV, MG, and NG (0.2%, *n* = 2). Single STI infection was present in 23.7% (222/935) of participants.

### 3.4. Multivariate Logistic Regression Analyses

Logistic regression analyses are summarized in Table 2. In multivariate logistic regression analyses, CT infection emerged as the strongest and most consistent predictor of HR-HPV across all models, remaining independently associated with overall HR-HPV [aOR: 4.52; 95% CI: 1.26–16.11; *p* = 0.019], Gardasil-9^®^-targeted genotype (aOR: 9.73; 95% CI: 2.72–34.86; *p* < 0.001), multiple HR-HPV infections (aOR: 13.96; 95% CI: 3.70–52.64; *p* < 0.001), and multiple Gardasil-9^®^-targeted genotypes (aOR: 15.29; 95% CI: 2.87–81.28; *p* = 0.001). MH infection was significantly associated with overall HR-HPV (aOR: 1.83; 95% CI: 1.13–2.47; *p* = 0.009) and nonvaccine HR-HPV (aOR: 2.75; 95% CI: 1.21–6.31; *p* = 0.016). UP infection was significantly associated with overall HR-HPV (aOR: 1.67; 95% CI: 1.13–2.47; *p* = 0.009), Gardasil-9^®^-targeted HR-HPV (aOR: 2.02; 95% CI: 1.25–3.25; *p* = 0.003), multiple HR-HPV (aOR: 2.33; 95% CI: 1.24–4.41; *p* = 0.008), multiple Gardasil-9^®^-targeted genotypes (aOR: 2.86; 95% CI: 1.03–7.90; *p* = 0.042), and multiple nonvaccine HR-HPV (aOR: 5.03; 95% CI: 1.56–16.19; *p* = 0.006). UU infection was associated with overall HR-HPV (aOR: 2.58; 95% CI: 1.22–5.48; *p* = 0.013).

Age was strongly associated with several HPV outcomes, with the age group [18–29] showing the highest overall HR-HPV prevalence [23.1% (48/208); *p* = 0.003], multiple HR-HPVs (8.2%, 17/208; *p* = 0.0004), both Gardasil-4^®^- and Gardasil-9^®^-targeted HR-HPV (7.7%, 16/208; *p* = 0.0016, and 14.9%, 31/208; *p* = 0.00054, respectively), and nonvaccine HR-HPV (13.9%, 29/208; *p* = 0.017).

HPV-16 and HPV-51 were significantly more detected in the age group [18–29] (6.2% 13/208, *p* = 0.011, and 5.9% 12/208, *p* = 0.006, respectively). Conversely, women aged [30–59] showed significantly reduced Odds ratios for HR-HPV infection (aOR: 0.41–0.55; *p* < 0.03) compared to those aged between 18 and 29 years.

The age-specific peaks for STIs were CT [1.0% (2/205)] and UP [29.7% (61/205)] for the age group [18–29], MH [5.7% (13/228)] for the age group [30–39], TV [0.8% (2/248)] for the age group [40–49], and UU [4.8% (11/230)] for the age group [50–59]. For STIs, UP was associated with the age group [18–29] (*p* = 0.00005), and MH with the age group [30–39] (*p* = 0.022).

In χ^2^-analyses by recruitment sites, no HR-HPV or STI outcome showed a significant side effect.

## 4. Discussion

This study demonstrates the successful implementation of a community-based screening program for HR-HPV and STIs among adult women in Romania, utilizing an innovative and secure digital and logistical workflow.

The study revealed the feasibility associated with logistical innovation of veil-based self-sampling. The primary observation lies in the demonstrated feasibility of the V-Veil UP2™ self-collection device within a large community setting. The logistical workflow was highly original, integrating a dedicated digital platform for participant registration and result communication. Despite the significant geographical distance between the collection sites in Romania and the accredited reference lab (Laboratoire Labio) in France, the process remained robust. This success is attributed to the high stability of the impregnated veil at room temperature, which allowed for the secure transport of samples via standard courier services (UPSs) without the need for cold-chain management, as previously demonstrated for other self-sampling devices [29,30,31].

The technical reliability of this method was confirmed by the very high rate of internal control positivity (98.2%; 943/960) (β-globin or internal STIs controls), indicating adequate cellularity and absence of PCR inhibitors in the collected samples. Previous research has similarly validated the analytical stability of dry-collected self-sampling devices for several weeks at ambient temperature, supporting their use in remote or international screening programs [30].

Concerning the HR-HPV and STI prevalence and genotypes, the study revealed a significant burden of infection, with an overall HR-HPV prevalence of 17.2% and an STI prevalence of 26.7% among participants with valid results. The most frequent HR-HPV genotypes were HPV-68 (3.0%), HPV-16 (2.9%), and HPV-31 (2.3%). Notably, the age group between 18 and 29 years exhibited the highest risk, with a prevalence of 22.2% for HR-HPV and 36.8% for any STI. The 18–29 age group emerged as the highest-risk group, showing the highest prevalence for overall HR-HPV, multiple HR-HPV infections, and specific high-risk HR-HPV genotypes like HPV-16. These findings strongly suggest that screening and vaccination efforts should be intensified in this demographic, potentially starting at younger ages. The significantly reduced Odds ratios for HR-HPV in women ≥ 30 years (aOR: 0.41–0.55) align with known HR-HPV epidemiology, where prevalence typically peaks shortly after sexual debut and then declines [32,33].

A critical finding regarding the Gardasil-9^®^ vaccine is that while its target genotypes were present in 9.6% of women, nonvaccine HR-HPV types accounted for over half (51.3%) of all HR-HPV infections detected. This underscores the ongoing necessity for comprehensive screening even in vaccinated populations [34]. Thus, while the Gardasil-9^®^ vaccine targets a significant portion of HR-HPV infections, the prevalence of nonvaccine HR-HPV types is even higher. This emphasizes that while vaccination is vital for primary prevention (especially against HPV-16), it must be complemented by robust screening to detect cervical disease caused by nonvaccine types.

Regarding STIs, UP was the most common pathogen (24.0%), followed by UU (4.5%) and MH (3.9%). These observations highlight the need for better surveillance and management of non-classical STIs, which are often overlooked in standard STI panels.

The multivariate analysis identified powerful associations between specific STIs and HR-HPV outcomes. CT emerged as the strongest and most consistent predictor, significantly increasing the Odds ratios for overall HR-HPV infection (aOR: 4.52), multiple HR-HPV infections (aOR: 13.96), as well as infection with vaccine-covered types (aOR: 10.60). These results align with the literature suggesting that CT infection may facilitate HPV acquisition or persistence by inducing local immunomodulation and mucosal damage [35,36,37,38,39].

Furthermore, MH and *Ureaplasma* species were independently associated with HR-HPV, particularly with nonvaccine types (MH, UP) or Gardasil-9^®^-targeted genotypes (UP), suggesting their role as potential cofactors in the microenvironment of the cervix [40,41,42]. These co-infections may promote the acquisition or persistence of HR-HPV, particularly genotypes not targeted by current vaccines, and should be considered during clinical workups. Indeed, MH and *Ureaplasma* species (such as UP or UU) disrupt the cervical microenvironment, facilitate HR-HPV persistence, and increase the risk of cervical disease—especially for nonvaccine types [43,44,45].

Taken together, these observations have direct implications for medical care and public health strategies in Romania, where cervical cancer mortality remains among the highest in Europe [46]. Firstly, the strong link between CT and HR-HPV suggests that parallel, integrated testing for both pathogens should be considered, particularly for women under 40. Screening and treatment for CT in cervical cancer screening programs (or vice versa) is strongly justified [37,38,47]. Finding a CT infection should immediately raise the clinical suspicion for an active, persistent, or multiple HR-HPV infection. Secondly, public health interventions and vaccination catch-up programs should target and prioritize the [18–29] age group, where infection rates peak. Finally, the success of veil-based self-sampling and digital result management offers a scalable model to reach underscreened populations who may face geographical or psychological barriers to traditional pelvic examinations and expand access to primary HPV molecular testing.

Although this pilot feasibility study yields highly promising results regarding the implementation of veil-based self-sampling, several limitations must be acknowledged. First, because the study population was prospectively invited from public and private gynecological centers, the cohort may inherently exhibit selection bias, potentially overrepresenting women who already have some degree of healthcare-seeking behavior or access to medical facilities. Consequently, these findings may not fully reflect the true screening adherence or epidemiological distribution of HR-HPV and STIs among the most vulnerable, isolated, or hard-to-reach rural populations in Romania who do not access clinical centers. It is likely that the study has not yet fully investigated the “underscreened” population living in Romania. Second, although the online digital platform facilitated participant registration and demographic data collection, it may have simultaneously created a digital literacy barrier. This could have limited independent participation, mostly among older women or those living in socioeconomically disadvantaged areas with poor internet access [48]. Third, the cross-sectional, prospective design of this pilot investigation precludes the longitudinal evaluation of viral persistence or the clinical progression of detected HR-HPV genotypes and concurrent STIs into high-grade cervical intraepithelial neoplasia or invasive cervical cancer. Thus, our pilot feasibility study was not designed to track clinical outcomes over time. Fourth, self-sampling within the framework of a protocol was offered through a healthcare facility, which may have facilitated its acceptance and use. Finally, while the multiplex real-time PCR assays provided high analytical sensitivity and specificity for the targeted pathogens, cytologic or histologic confirmation was not performed in parallel for all participants within the scope of this baseline screening protocol. Future large-scale, population-based longitudinal studies are warranted to validate the long-term clinical efficacy and cost-effectiveness of integrating this digital, veil-based self-sampling workflow into the national cervical cancer screening framework.

## 5. Conclusions

This pilot study confirms that veil-based self-collection combined with a digital logistics platform is a highly feasible, effective, and community-friendly method for cervical cancer and STI screening in Romania. This method can significantly improve participation, especially in underserved or harder-to-reach populations. The high stability of the samples at room temperature enables centralized testing in specialized laboratories, regardless of distance. Clinically, the dramatic association between CT and HR-HPV, as well as the strong association between MH and *Ureaplasma* species and HR-HPV, highlights a critical need for co-testing and integrated management. Future public health efforts must leverage these non-invasive tools to increase screening coverage and address the significant burden of nonvaccine HR-HPV types and associated STIs in the female community.

## Figures and Tables

**Table 1:** Age and distribution of HPV types and essential STIs in the study’s 960 adult women according to their inclusion sites in Romania.

Characteristics	All Participants [*n* = 960]	Inclusion Sites							
		Braila [*n* = 25]	Bucuresti [*n* = 76]	Constanta [*n* = 241]	Craiova [*n* = 56]	Galati [*n* = 23]	Iasi [*n* = 370]	Pitesti [*n* = 12]	Suceava [*n* = 157]
Age [*n* (%)]									
All ages [mean (SD); year]	41.5 (11.7)	44.0 (11.0)	43.8 (11.8)	42.3 (10.2)	40.1 (11.8)	41.5 (12.2)	39.3 (12.8)	40.8 (12.8)	44.4 (10.0)
[18–29]	202 (21.0%)	4 (16.0%)	11 (14.5%)	31 (12.9%)	14 (25.0%)	6 (26.1%)	117 (31.6%)	2 (16.7%)	17 (10.8%)
[30–39]	234 (24.4%)	3 (12.0%)	16 (21.0%)	79 (32.8%)	15 (26.8%)	4 (17.4%)	76 (20.5%)	4 (33.3%)	37 (23.6%)
[40–49]	244 (25.4%)	8 (32.0%)	17 (22.4%)	63 (26.1%)	14 (25.0%)	6 (26.1%)	91 (24.7%)	2 (16.7%)	43 (27.4%)
[50–59]	244 (25.4%)	10 (40.0%)	29 (38.2%)	64 (26.6%)	10 (17.8%)	6 (26.1%)	67 (18.1%)	4 (33.3%)	54 (34.4%)
≥60	36 (3.8%)	0 (0.0%)	3 (3.9%)	4 (1.7%)	3 (5.4%)	1 (4.3%)	19 (5.1%)	0 (0.0%)	6 (3.8%)
HPV DNA detection and genotypes [*n* = 938 participants positive for β-globin internal control of HR-HPV kit] [*n* (%) [95% CI] *]									
HR-HPV DNA	161 (17.3%) [15.0%, 20.0%]	2 (8.0%) [1.4%, 28.0%]	12 (16.2%) [9.0%, 27.0%]	46 (19.9%) [15.0%, 26.0%]	13 (23.2%) [13.0%, 37.0%]	3 (13.6%) [3.6%, 36.0%]	63 (17.4%) [14%, 22.0%]	3 (25.0%) [6.7%, 57.0%]	19 (12.9%) [8.2%, 20.0%]
Multiple (≥2) HR-HPV	45 (4.8%) [3.6%, 6.5%]	2 (8.0%) [1.4%, 28.0%]	4 (5.4%) [1.7%, 14.0%]	9 (3.9%) [1.9%, 7.5%]	3 (5.4%) [1.4%, 16.0%]	1 (4.5%) [0.2%, 25.0%]	18 (5.0%) [3.1%, 7.9%]	2 (16.7%) [2.9%, 49.0%]	6 (4.1%) [1.7%, 9.1%]
HPV-16	27 (2.9%) [2.0%, 4.3%]	0 (0.0%) [0.0%, 17.0%]	4 (5.4%) [1.7%, 14.0%]	4 (1.7%) [0.56%, 4.7%]	1 (1.8%) [0.0%, 11%]	0 (0.0%) [0.0%, 18.0%]	15 (4.1%) [2.4%, 6.9%]	1 (8.3%) [0.4%, 40.0%]	2 (1.4%) [0.2%, 5.3%]
HPV-18	11 (1.2%) [0.6%, 2.2%]	0 (0.0%) [0.0%, 17.0%]	0 (0.0%) [0.0%, 6.1%]	1 (0.4%) [0.0%, 2.8%]	2 (3.6%) [0.6%, 13%]	0 (0.0%) [0.0%, 18.0%]	6 (1.7%) [0.67%, 3.7%]	0 (0.0%) [0.0%, 30%]	2 (1.4%) [0.2%, 5.3%]
HPV-31	22 (2.4%) [1.5%, 3.6%]	0 (0.0%) [0.0%, 17%]	1 (1.4%) [0.0%, 8.3%]	8 (3.5%) [1.6%, 7.0%]	1 (1.8%) [0.09%, 11.0%]	1 (4.5%) [0.24%, 25%]	5 (1.4%) [0.51%, 3.4%	2 (16.7%) [2.9%, 49.0%]	4 (2.7%) [0.8%, 7.3%]
HPV-33	10 (1.1%) [0.5%, 2.0%]	1 (4.0%) [0.2%, 22.0%]	1 (1.4%) [0.0%, 8.3%]	2 (0.9%) [0.1%, 3.4%]	0 (0.0%) [0.0%, 8.0%]	0 (0.0%) [0.0%, 18.0%]	5 (1.4%) [0.5%, 3.4%]	0 (0.0%) [0.0%, 30.0%]	1 (0.7%) [0.0%, 4.3%]
HPV-35	6 (0.6%) [0.2%, 1.5%]	1 (4.0%) [0.2%, 22.0%]	0 (0.0%) [0.0%, 6.1%]	1 (0.4%) [0.0%, 2.8%]	0 (0.0%) [0.0%, 8.0%]	0 (0.0%) [0.0%, 18.0%]	3 (0.8%) [0.21%, 2.6%]	0 (0.0%) [0.0%, 30%]	1 (0.7%) [0.0%, 4.3%]
HPV-39	18 (1.9%) [1.2%, 3.1%]	0 (0.0%) [0.0%, 17.0%]	1 (1.4%) [0.0%, 8.3%]	6 (2.6%) [1.1%, 5.8%]	2 (3.6%) [0.6%, 13.0%]	0 (0.0%) [0.0%, 18.0%]	7 (1.9%) [0.8%, 4.1%]	0 (0.0%) [0.0%, 30.0%]	2 (1.4%) [0.2%, 5.3%]
HPV-45	11 (1.2%) [0.6%, 2.2%]	0 (0.0%) [0.0%, 17.0%]	0 (0.0%) [0.00%, 6.1%]	3 (1.3%) [0.3%, 4.1%]	0 (0.0%) [0.0%, 8.0%]	0 (0.0%) [0.0%, 18.0%]	5 (1.4%) [0.5%, 3.4%]	1 (8.3%) [0.4%, 40.0%]	2 (1.4%) [0.2%, 5.3%]
HPV-51	20 (2.2%) [1.4%, 3.4%]	0 (0.0%) [0.0%, 17.0%]	1 (1.4%) [0.0%, 8.3%]	5 (2.2%) [0.8%, 5.3%]	1 (1.8%) [0.0%, 11.0%]	0 (0.0%) [0.0%, 18.0%]	11 (3.0%) [1.6%, 5.5%]	0 (0.0%) [0.0%, 30%]	2 (1.4%) [0.2%, 5.3%]
HPV-52	18 (1.9%) [1.2%, 3.1%]	0 (0.0%) [0.0%, 17.0%]	1 (1.4%) [0.0%, 8.3%]	2 (0.9%) [0.1%, 3.4%]	2 (3.6%) [0.6%, 13.0%]	0 (0.0%) [0.0%, 18.0%]	9 (2.5%) [1.2%, 4.8%]	1 (8.3%) [0.4%, 40.0%]	3 (2.0%) [0.5%, 6.3%]
HPV-56	18 (1.9%) [1.2%, 3.1%]	0 (0.0%) [0.0%, 17.0%]	2 (2.7%) [0.4%, 10.0%]	3 (1.3%) [0.3%, 4.1%]	3 (5.4%) [1.4%, 16.0%]	2 (9.1%) [1.6%, 31.0%]	7 (1.9%) [0.8%, 4.1%]	0 (0.0%) [0.0%, 30.0%]	1 (0.7%) [0.0%, 4.3%]
HPV-58	12 (1.3%) [0.7%, 2.3%]	0 (0.0%) [0.0%, 17.0%]	1 (1.4%) [0.0%, 8.3%]	2 (0.9%) [0.1%, 3.4%]	0 (0.0%) [0.0%, 8.0%]	0 (0.0%) [0.0%, 18.0%]	7 (1.9%) [0.8%, 4.1%]	0 (0.0%) [0.0%, 30.0%]	2 (1.4%) [0.2%, 5.3%]
HPV-59	7 (0.8%) [0.3%, 1.6%]	0 (0.0%) [0.0%, 17.0%]	0 (0.0%) [0.0%, 6.1%]	2 (0.9%) [0.1%, 3.4%]	2 (3.6%) [0.6%, 13.0%]	0 (0.0%) [0.0%, 18.0%]	3 (0.8%) [0.2%, 2.6%]	0 (0.0%) [0.0%, 30.0%]	0 (0.0%) [0.0%, 3.2%]
HPV-66	21 (2.3%) [1.4%, 3.5%]	1 (4.0%) [0.21%, 22.0%]	5 (6.8%) [2.5%, 16.0%]	7 (3.0%) [1.3%, 6.4%]	1 (1.8%) [0.0%, 11.0%]	0 (0.0%) [0.0%, 18.0%]	4 (1.1%) [0.35%, 3.0%]	1 (8.3%) [0.4%, 40.0%]	2 (1.4%) [0.2%, 5.3%]
HPV-68	28 (3.0%) [2.0%, 4.4%]	1 (4.0%) [0.2%, 22.0%]	1 (1.4%) [0.0%, 8.3%]	11 (4.8%) [2.5%, 8.6%]	2 (3.6%) [0.6%, 13.0%]	1 (4.5%) [0.2%, 25.0%]	8 (2.2%) [1.0%, 4.5%]	0 (0.0%) [0.0%, 30.0%]	4 (2.7%) [0.88%, 7.3%]
Any 4-valent vaccine types **	35 (3.8%) [2.7%, 5.3%]	0 (0.0%) [0.0%, 17.0%]	4 (5.4%) [1.7%, 14.0%]	5 (2.2%) [0.80%, 5.3%]	3 (5.4%) [1.4%, 16.0%]	0 (0.0%) [0.0%, 18.0%]	18 (5.0%) [3.1%, 7.9%]	1 (8.3%) [0.44%, 40.0%]	4 (2.7%) [0.8%, 7.3%]
Multiple 4-valent vaccine types	3 (0.3%) [0.0%, 1.0%]	0 (0.0%) [0.0%, 17.0%]	0 (0.0%) [0.0%, 6.1%]	0 (0.0%) [0.0%, 2.0%]	0 (0.0%) [0.0%, 8.0%]	0 (0.0%) [0.0%, 18.0%]	3 (0.8%) [0.2%, 2.6%]	0 (0.0%) [0.0%, 30.0%]	0 (0.0%) [0.0%, 3.2%]
Any 9-valent vaccine types ***	90 (9.7%) [7.9%, 12.0%]	1 (4.0%) [0.21%, 22.0%]	7 (9.5%) [4.2%, 19.0%]	20 (8.7%) [5.5%, 13.0%]	6 (10.7%) [4.4%, 23.0%]	1 (4.5%) [0.24%, 25.0%]	39 (10.7%) [7.8%, 15.0%]	3 (25.0%) [6.7%, 57.0%]	13 (8.8%) [5.0%, 15.0%]
Multiple 9-valent vaccine types	16 (1.7%) [1.0%, 2.8%]	0 (0.0%) [0.0%, 17%]	1 (1.4%) [0.0%, 8.3%]	2 (0.9%) [0.1%, 3.4%]	0 (0.0%) [0.0%, 8.0%]	0 (0.0%) [0.0%, 18.0%]	9 (2.5%) [1.2%, 4.8%]	1 (8.3%) [0.4%, 40.0%]	3 (2.0%) [0.5%, 6.3]
Nonvaccine HR-HPV types	105 (11.3%) [9.4%, 14.0%]	2 (8.0%) [1.4%, 28.0%]	8 (10.8%) [5.1%, 21.0%]	33 (14.3%) [10%, 20.0%]	10 (17.9%) [9.3%, 31.0%]	3 (13.6%) [3.6%, 36.0%]	38 (10.5%) [7.6%, 14.0%]	1 (8.3%) [0.4%, 40.0%]	10 (6.8%) [3.5%, 12.0%]
Sexually transmitted infections [*n* = 935 participants positive for internal control of STIs kit] [*n* (%) [95% CI] *]									
Any STIs	248 (26.7%) {24.0%, 30.0%}	7 (28.0%) [13.0%, 50.0%]	19 (25.7%) [17.0%, 37.0%]	63 (27.3%) [22.0%, 34.0%]	18 (32.1%) [21%, 46.0%]	4 (18.2%) [6.0%, 41.0%]	97 (26.7%) [22.0%, 32.0%]	2 (16.7%) [2.9%, 49.0%]	38 (25.9%) [19%, 34.0%]
*C. trachomatis*	10 (1.1%) [0.5%, 2.0%]	1 (4.0%) [0.2%, 22.0%]	1 (1.4%) [0.0%, 8.3%]	0 (0.0%) [0.0%, 2.0%]	0 (0.0%) [0.0%, 8.0%]	0 (0.0%) [0.0%, 18.0%]	5 (1.4%) [0.5%, 3.4%]	1 (8.3%) [0.4%, 40%]	2 (1.4%) [0.2%, 5.3%]
*N. gonorrhoeae*	2 (0.2%) [0.0%, 0.8%]	0 (0.0%) [0.0%, 17.0%]	0 (0.0%) [0.0%, 6.1%]	0 (0.0%) [0.0%, 2.0%]	0 (0.0%) [0.0%, 8.0%]	0 (0.0%) [0.0%, 18.0%]	0 (0.0%) [0.0%, 1.3%]	0 (0.0%) [0.0%, 30.0%]	2 (1.4%) [0.2%, 5.3%]
*M. genitalium*	2 (0.2%) [0.0%, 0.86%]	0 (0.0%) [0.0%, 17.0%]	0 (0.0%) [0.0%, 6.1%]	0 (0.0%) [0.0%, 2.0%]	1 (1.8%) [0.0%, 11.0%]	0 (0.0%) [0.0%, 18.0%]	0 (0.0%) [0.0%, 1.3%]	0 (0.0%) [0.0%, 30.0%]	1 (0.7%) [0.0%, 4.3%]
*M. hominis*	32 (3.4%) [2.4%, 4.9%]	1 (4.0%) [0.2%, 22.0%]	0 (0.0%) [0.0%, 6.1%]	16 (6.9%) [4.1%, 11.0%]	2 (3.6%) [0.6%, 13.0%]	0 (0.0%) [0.0%, 18.0%]	9 (2.5%) [1.2%, 4.8%]	0 (0.0%) [0.0%, 30%]	4 (2.7%) [0.8%, 7.3%]
*T. vaginalis*	2 (0.2%) [0.0%, 0.8%]	1 (4.0%) [0.2%, 22.0%]	0 (0.0%) [0.0%, 6.1%]	1 (0.4%) [0.0%, 2.8%]	0 (0.0%) [0.0%, 8.0%]	0 (0.0%) [0.0%, 18.0%]	0 (0.0%) [0.0%, 1.3%]	0 (0.0%) [0.0%, 30.0%]	0 (0.0%) [0.0%, 3.2%]
*U. parvum*	198 (21.3%) [19%, 24.0%]	4 (16.0%) [5.3%, 37.0%]	16 (21.6%) [13%, 33.0%]	45 (19.5%) [15%, 25.0%]	15 (26.8%) [16%, 41.0%]	3 (13.6%) [3.6%, 36.0%]	80 (22.0%) [18%, 27.0%]	1 (8.3%) [0.4%, 40.0%]	34 (23.1%) [17.0%, 31.0%]
*U. urealyticum*	34 (3.7%) [2.6%, 5.1%]	1 (4.0%) [0.2%, 22.0%]	3 (4.1%) [1.1%, 12.0%]	11 (4.8%) [2.5%, 8.6%]	2 (3.6%) [0.62%, 13.0%]	1 (4.5%) [0.24%, 25.0%]	11 (3.0%) [1.6%, 5.5%]	0 (0.0%) [0.0%, 30.0%]	5 (3.4%) [1.3%, 8.2%]

* The 95% confidence intervals are presented in brackets. ** The 4-valent Gardasil-4^®^ vaccine (Merck & Co. Inc., Rahway, NJ, USA) is effective against HPV genotypes -6, -11, -16, and -18. *** The 9-valent Gardasil-9^®^ vaccine (Merck & Co. Inc.) is effective against HPV genotypes -6, -11, -16, -18, 31, -33, -45, -52, and -58. HPV: Human papillomavirus. HR-HPV: High-risk human papillomavirus. SD: Standard deviation. STI: Sexually Transmitted Infection.

**Table 2:** Demographic and infectious predictors of HR-HPV outcomes by univariate and multivariate logistic regression analyses.

	HR-HPV				Gardasil-9^®^-Vaccine-HR-HPV				Nonvaccine HR-HPV				Multiple HR-HPV				Multiple Gardasil-9^®^-Vaccine-HR-HPV				Multiple Nonvaccine HR-HPV			
	cOR (95% CI)	*p*	aOR (95% CI)	*p*	cOR (95% CI)	*p*	aOR (95% CI)	*p*	cOR (95% CI)	*p*	aOR (95% CI)	*p*	cOR (95% CI)	*p*	aOR (95% CI)	*p*	cOR (95% CI)	*p*	aOR (95% CI)	*p*	cOR (95% CI)	*p*	aOR (95% CI)	*p*
Age (years)																								
[18–29]	REF	REF	REF	REF	REF	REF	REF	REF	REF	REF	REF	REF	REF	REF	REF	REF	REF	REF	REF	REF	REF	REF	REF	REF
[30–39]	1.05 (0.69–1.62)	0.404	0.55 (0.33–0.94)	0.027	0.85 (0.34–2.13)	0.733	NA	NA	1.02 (0.59–1.72)	0.956	NA	NA	0.74 (0.31–1.84)	0.527	NA	NA	0.00 (0.00–Inf)	0.998	NA	NA	4.76 (0.79–28.66)	0.088	NA	NA
[40–49]	0.59 (0.37–0.95)	0.030	0.45 (0.26–0.78)	0.004	0.33 (0.10–1.12)	0.075	NA	NA	0.62 (0.34–1.11)	0.103	NA	NA	0.54 (0.21–1.42)	0.213	NA	NA	1.07 (0.28–4.07)	0.917	NA	NA	0.00 (0.00–Inf)	0.998	NA	NA
[50–59]	0.63 (0.39–1.02)	0.057	0.41 (0.23–0.72)	0.002	0.51 (0.17–1.12)	0.228	NA	NA	0.63 (0.34–1.14)	0.125	NA	NA	0.33 (0.09–1.09)	0.070	NA	NA	0.7 (0.15–3.21)	0.649	NA	NA	0.00 (0.00–Inf)	0.998	NA	NA
≥60	0.83 (0.29–2.42)	0.741	0.63 (0.21–1.95)	0.425	0.00 (0.00–Inf)	0.999	NA	NA	1.04 (0.31–3.49)	0.944	NA	NA	0.00 (0.00–Inf)	0.998	NA	NA	0.00 (0.00–Inf)	0.998	NA	NA	0.00 (0.00–Inf)	0.998	NA	NA
Inclusion																								
Bucuresti	REF	REF	REF	REF	REF	REF	REF	REF	REF	REF	REF	REF	REF	REF	REF	REF	REF	REF	REF	REF	REF	REF	REF	REF
Iasi	1.08 (0.55–2.13)	0.812	NA	NA	1.12 (0.47–2.61)	0.795	NA	NA	0.96 (0.43–2.16)	0.930	NA	NA	0.91 (0.29–2.78)	0.873	NA	NA	1.85 (0.23–14.87)	0.561	NA	NA	0.40 (0.07–2.23)	0.296	NA	NA
Pitesti	1.72 (0.41–7.31)	0.461	NA	NA	3.19 (0.69–14.61)	0.135	NA	NA	0.75 (0.08–6.59)	0.795	NA	NA	3.50 (0.56–21.64)	0.177	NA	NA	6.63 (0.38–113.97)	0.192	NA	NA	1.16 (0.05–25.62)	0.925	NA	NA
Craiova	1.56 (0.65–3.75)	0.318	NA	NA	1.15 (0.36–3.63)	0.813	NA	NA	1.79 (0.65–4.88)	0.253	NA	NA	0.99 (0.21–4.61)	0.991	NA	NA	0.43 (0.02–10.84)	0.611	NA	NA	0.65 (0.05–7.41)	0.732	NA	NA
Suceava	0.81 (0.37–1.77)	0.603	NA	NA	0.93 (0.35–2.43)	0.880	NA	NA	0.60 (0.23–1.59)	0.307	NA	NA	0.74 (0.20–2.72)	0.656	NA	NA	1.52 (0.15–14.87)	0.718	NA	NA	0.49 (0.06–3.59)	0.488	NA	NA
Constanta	1.27 (0.63–2.56)	0.491	NA	NA	0.91 (0.36–2.23)	0.824	NA	NA	1.36 (0.60–3.11)	0.454	NA	NA	0.71 (0.21–2.36)	0.572	NA	NA	0.63 (0.05–7.10)	0.712	NA	NA	0.31 (0.04–2.26)	0.249	NA	NA
Galati	0.815 (0.21–3.19)	0.771	NA	NA	0.45 (0.05–3.92)	0.474	NA	NA	1.30 (0.31–5.39)	0.715	NA	NA	0.83 (0.08–7.86)	0.873	NA	NA	1.08 (0.04–27.67)	0.958	NA	NA	0.64 (0.03–13.92)	0.779	NA	NA
Braila	0.45 (0.09–2.16)	0.318	NA	NA	0.39 (0.04–3.41)	0.401	NA	NA	0.72 (0.14–3.63)	0.687	NA	NA	1.52 (0.26–8.85)	0.640	NA	NA	0.96 (0.03–24.34)	0.981	NA	NA	1.50 (0.13–17.28)	0.745	NA	NA
Sexually transmitted infections																								
*C. trachomatis*	4.86 (1.39–17.01)	0.013	4.52 (1.26–16.11)	0.019	9.96 (2.83–35.12)	0.0003	9.73 (2.72–34.86)	0.0004	3.44 (0.87–13.52)	0.076	NA	NA	14.31 (3.88–52.68)	0.00006	13.96 (3.70–52.64)	0.00009	16.19 (3.15–83.24)	0.0008	15.29 (2.87–81.28)	0.001	9.19 (1.07–78.90)	0.043	7.06 (0.77–64.28)	0.083
*N. gonorrhoeae*	4.77 (0.29–76.66)	0.270	NA	NA	9.55 (0.59–154.13)	0.112	NA	NA	1.56 (0.07–32.78)	0.773	NA	NA	3.88 (0.18–82.17)	0.383	NA	NA	11.07 (0.51–239.77)	0.125	NA	NA	14.68 (0.67–321.75)	0.088	NA	NA
*M. genitalium*	0.94 (0.04–19.77)	0.971	NA	NA	1.87 (0.08–39.43)	0.684	NA	NA	1.56 (0.07–32.78)	0.773	NA	NA	3.88 (0.18–82.17)	0.383	NA	NA	11.07 (0.51–239.77)	0.125	NA	NA	14.68 (0.67–321.75)	0.088	NA	NA
*M. hominis*	2.59 (1.23–5.49)	0.012	1.83 (1.13–2.47)	0.009	1.79 (0.67–4.78)	0.241	NA	NA	2.75 (1.21–6.31)	0.016	2.75 (1.21–6.31)	0.016	2.11 (0.62–7.21)	0.233	NA	NA	0.82 (0.05–14.03)	0.893	NA	NA	5.93 (1.24–28.24)	0.025	4.12 (0.83–20.35)	0.083
*T. vaginalis*	4.77 (0.29–76.66)	0.270	NA	NA	1.87 (0.08–39.43)	0.684	NA	NA	7.93 (0.49–127.78)	0.144	NA	NA	3.88 (0.18–82.17)	0.383	NA	NA	11.07 (0.51–239.77)	0.125	NA	NA	14.68 (0.67–321.75)	0.088	NA	NA
*U. parvum*	1.74 (1.18–2.54)	0.004	1.67 (1.13–2.47)	0.009	2.04 (1.27–3.26)	0.003	2.02 (1.25–3.25)	0.003	1.48 (0.93–2.35)	0.092	NA	NA	2.36 (1.26–4.42)	0.006	2.33 (1.24–4.41)	0.008	2.95 (1.08–8.02)	0.034	2.86 (1.03–7.90)	0.042	5.34 (1.67–16.99)	0.004	5.03 (1.56–16.19)	0.006
*U. urealyticum*	2.73 (1.32–5.64)	0.006	2.58 (1.22–5.48)	0.013	1.68 (0.63–4.48)	0.292	NA	NA	2.13 (0.91–5.03)	0.083	NA	NA	3.40 (1.13–10.19)	0.028	2.24 (0.62–8.13)	0.221	0.00 (0.00–Inf)	0.999	NA	NA	5.46 (0.59–49.96)	0.133	NA	NA

cOR: crude Odds ratio. aOR: adjusted Odds ratio. HR-HPV: High-Risk human papillomavirus. NA: Not attributable. REF: Reference category used for comparison in the logistic regression analysis. cORs and aORs for the other categories were estimated relative to this group.

## Data Availability

The raw data supporting the conclusions of this article will be made available by the authors on request.
